# Stress fracture of the scaphoid in an elite junior tennis player: a case report and review of the literature

**DOI:** 10.1186/s13256-015-0785-3

**Published:** 2016-01-18

**Authors:** Sho Kohyama, Akihiro Kanamori, Toshikazu Tanaka, Yuki Hara, Masashi Yamazaki

**Affiliations:** 1 Department of Orthopedic Surgery, Faculty of Medicine, University of Tsukuba, 1-1-1 Tennodai, Tsukuba, Ibaraki 305-8576 Japan; 2 Department of Orthopedic Surgery, Kikkoman General Hospital, 100 Miyazaki, Noda, Chiba 278-0005 Japan

**Keywords:** Carpal scaphoid, Stress fracture, Tennis

## Abstract

**Background:**

The carpal scaphoid is the most commonly fractured carpal bone in young adults after a fall on an outstretched arm that results in acute dorsal flexion of the wrist. However, stress fractures of the scaphoid are relatively rare. To the best of our knowledge, we describe the first case in the literature of carpal scaphoid stress fracture in a tennis player.

**Case presentation:**

An 18-year-old Japanese man who was an elite junior tennis player was referred to our hospital after radiography and computed tomography revealed a carpal scaphoid fracture. The patient presented with pain in the wrist joint and tenderness over the anatomical snuff-box with diffuse swelling and reduced active dorsal flexion and flexion of the right wrist. The patient was treated conservatively and resumed participation in competitive events 5 months after his initial presentation.

**Conclusions:**

In this case, the scaphoid stress fracture had resulted from repetitive practicing of the attacking backhand high volley, which involved excessive dorsal flexion of the wrist. Although rare, scaphoid stress fractures must be considered in tennis players with chronic wrist pain.

## Background

Stress fracture of the scaphoid has been attributed to repeated dorsal flexion of the wrist [1]. Carpal scaphoid stress fractures have been reported in individuals engaging in gymnastics, shot put, diving, badminton, and cricket [1–10]. To the best of our knowledge, we report the first case in the literature of carpal scaphoid stress fracture in an elite junior tennis player. In our patient, the scaphoid stress fracture was a result of repetitive practice of the backhand high volley, which required excessive dorsal flexion of the wrist.

## Case presentation

An 18-year-old man who was an elite junior tennis player developed severe pain over the dorsal aspect of his right wrist when he hit a backhand volley in a tournament match. There was no major trauma, but he could not continue playing in the match and therefore visited a local clinic. On the basis of radiography and computed tomography (CT) findings, he was diagnosed with a carpal scaphoid fracture and was referred to our facility.

The patient is a right-handed player and mainly hits one-handed forehand and one-handed backhand shots. For 4 months before presentation, he had been repeatedly practicing his backhand volley, especially the attacking backhand high volley. He began to experience gradual pain in the right wrist, which worsened with wrist dorsal flexion. However, he had not visited any medical facility and had continued practicing tennis.

His physical examination revealed tenderness over the anatomical snuff-box, with diffuse swelling. The active dorsal flexion and volar flexion of the right wrist was 10 degrees less than in the left wrist, and forced passive dorsal flexion and volar flexion of the right wrist caused increased pain over the wrist joint. Pronation and supination of the forearm and digital motion were within normal limits.

**Fig. 1:**
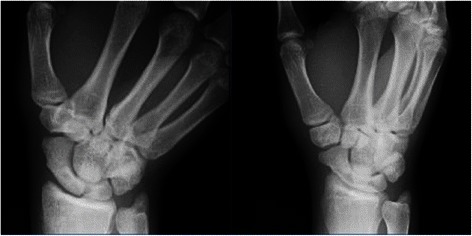
Radiographs obtained at the first visit show a nondisplaced scaphoid waist fracture through the carpal scaphoid with slightly increased bone density

**Fig. 2:**
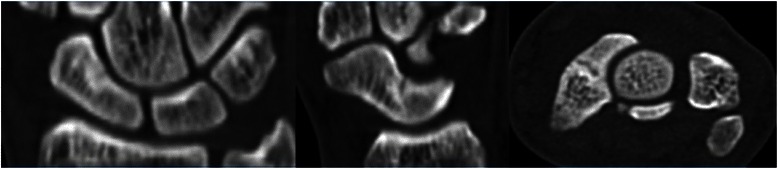
Computed tomographic image from the first visit shows a nondisplaced fracture at the scaphoid waist. Sclerotic and cystic changes suggest stress fractures of the scaphoid

Radiographs revealed a nondisplaced scaphoid waist fracture through the carpal scaphoid with slightly increased bone density at the edge of the fracture line (Fig. 1). CT also revealed a nondisplaced scaphoid fracture at its waist, and both sclerotic and cystic changes were observed (Fig. 2). This finding, together with the patient’s history, suggested stress fracture of the scaphoid.

**Fig. 3:**
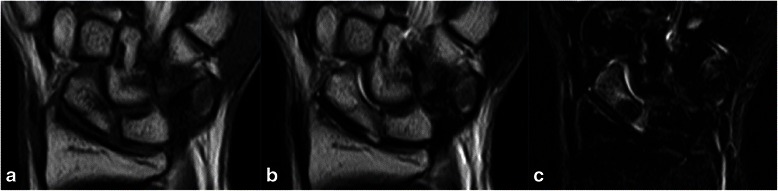
Magnetic resonance imaging scans obtained 2 weeks after the patient’s initial presentation. Low intensity can be seen on the T1-weighted image (**a**), isointensity to high intensity on the T2-weighted image (**b**), and low intensity on the T2-weighted image, with fat suppression (**c**) at the waist of the scaphoid

Magnetic resonance imaging performed 2 weeks after the patient’s initial presentation showed low intensity on the T1-weighted image, isointensity to high intensity on the T2-weighted image, and low intensity on the T2-weighted image, with fat suppression at the waist of the scaphoid (Fig. 3).

**Fig. 4:**
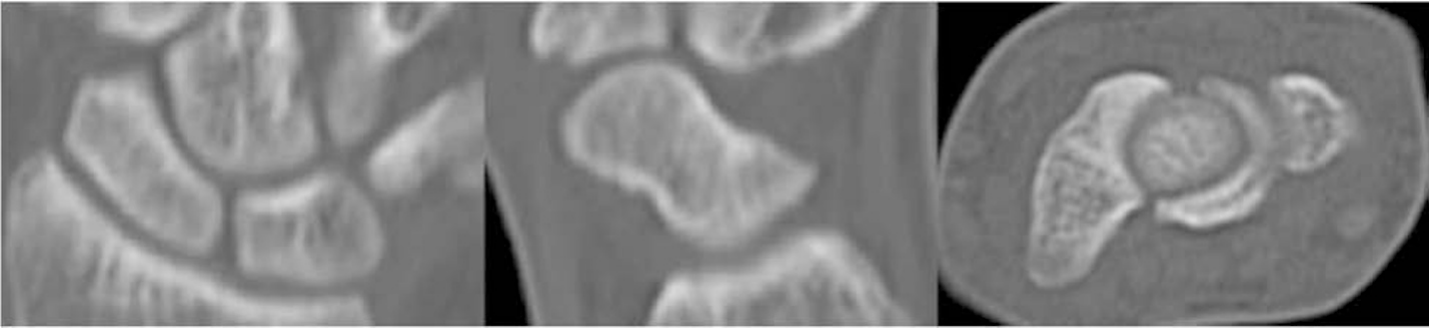
Computed tomography performed 3 months after the initial presentation. The image shows complete healing of the fracture

For treatment, we proposed either conservative therapy or surgery, and the patient chose to follow a course of conservative therapy. His right wrist was immobilized in a short thumb spica cast for 4 weeks, followed by a wrist brace (WRIST CARE PRO, ALCARE Co., Tokyo, Japan) for another 8 weeks. After cast immobilization, the patient was instructed, under the supervision of an occupational therapist, how to best perform active dorsal flexion and volar flexion. He was encouraged to perform this rehabilitation regimen at least three times daily without the wrist brace. Eight weeks after his initial presentation (4 weeks after cast immobilization), he displayed no tenderness over the anatomical snuff-box. Subsequently, he was instructed to perform passive dorsal flexion and volar flexion of the wrist. Three months after the patient’s initial presentation, a CT scan showed that the fracture had healed and the patient was asymptomatic (Fig. 4). He was allowed to gradually return to playing tennis, starting with forehand strokes that do not require dorsal flexion of the wrist on impact. When he was able to hit forehand strokes normally without any symptoms, he slowly began practicing backhand strokes. Five months after his initial presentation, he resumed participation in competitive events. At his final examination, 1 year after presentation, the range of motion of his wrist was normal and he had experienced no recurrence of wrist pain. The patient was completely satisfied with the result.

## Discussion

The carpal scaphoid is the most commonly fractured carpal bone [6]. Carpal scaphoid fractures usually occur in young adults after a fall on an outstretched arm that results in acute dorsal flexion of the wrist [9]. However, stress fractures of the scaphoid are relatively rare, and although such cases have been reported in relation to other sports [1–10], we believe this is the first report of carpal scaphoid fracture in a tennis player (Table 1).

**Table 1:** Previously reported stress fractures of the carpal scaphoid

Report	Patient age (yr), sex	Sport	Treatment	Period required to return to sports
Manzione and Pizzutillo, 1981 [2]	16, M	Gymnast	Conservative	10 wk
Hanks *et al*., 1989 [1]	19, M	Shot put (*n* = 1),	Conservative	12–32 wk
	18, M	Gymnastics (*n* = 3)		
	18, M			
	18, M			
Engel and Feldner-Busztin, 1991 [3]	18, M	Gymnastics	Not mentioned	Not mentioned
Inagaki and Inoue, 1997 [4]	16, M	Badminton	Conservative	11 wk
Matzkin and Singer, 2000 [5]	13, F	Gymnastics	Conservative	24 wk
Hosey *et al*., 2006 [6]	13, F	Diving	Internal fixation	Not mentioned
Rethnam *et al*., 2006 [7]	38, M	Cricket	Conservative	Not mentioned
Yamagiwa *et al*., 2009 [8]	18, M	Gymnastics	Conservative, internal fixation	8 wk
Nakamoto *et al*., 2011 [9]	18, M	Gymnastics	Internal fixation	12 wk
Pidemunt *et al*., 2012 [10]	13, M	Soccer goalkeeper	Internal fixation	24 wk
Our patient	18 M	Tennis	Conservative	20 wk

In a biomechanical study, Majima *et al.* [11] reported that loading patterns at the wrist are different in the dorsally flexed position than in the neutral position. When the wrist is placed in the dorsally flexed position, force transmission shifts radially and concentrates at the scaphoid [11]. Extreme dorsal flexion compresses the radioscaphoid articulation and drives the scaphoid to the volar side. The volar radioscapholunate and radiocapitate ligaments resist the volar force from the proximal scaphoid. Distal to these are less restrictive radiocollateral ligaments, which make the scaphoid just distal to the proximal volar ligaments biomechanically the weakest point [5]. This is where the fracture line was observed in our patient: the waist of the scaphoid.

Modern tennis players require a wide range of wrist movements for the various strokes and volleys. In backhand strokes and backhand volleys, the wrist is in neutral to 2–5 degrees of radial deviation and requires dorsal flexion of the wrist upon impact, especially in advanced players [12]. Chow *et al.* reported that muscle activation of the extensor carpi radialis in the backhand volley was greater than for the forehand volley in both the pre- and postimpact phases [13]. This suggests that dorsal flexion plays an important role in the backhand volley.

We concluded that the initial cause of the stress fracture in our patient was excessive and repetitive practice of the attacking backhand high volley. This exertion resulted in the carpal scaphoid receiving repetitive shearing and torsional forces, causing a stress fracture at the scaphoid waist. To prevent injury recurrence, the patient was advised to change the form of the backhand volley and not to dorsally flex the wrist excessively. Because the attacking backhand high volley is a relatively less necessary shot in tennis, the patient was advised not to overpractice the shot.

## Conclusions

To the best of our knowledge, this is the first reported case in the literature of carpal scaphoid stress fracture in a tennis player. Repetitive practice of the backhand volley, which requires excessive dorsal flexion of the wrist, led to a stress fracture of the carpal scaphoid. A sound bony union was obtained with conservative therapy, and the patient resumed participating in competitive tennis within 5 months of initial presentation. Although scaphoid stress fracture is comparatively rare, we suggest that it must be considered in the differential diagnosis when tennis players present with chronic wrist pain.

## Consent

Written informed consent was obtained from the patient for publication of this case report and any accompanying images. A copy of the written consent is available for review by the Editor-in-Chief of this journal.

## References

[1] Hanks GA, Kalenak A, Bowman LS, Sebastianelli WJ. Stress fractures of the carpal scaphoid: a report of four cases. J Bone Joint Surg Am. 1989;71:938–41. 2745491

[2] Manzione M, Pizzutillo PD. Stress fracture of the scaphoid waist: a case report. Am J Sports Med. 1981;9:268–9. 10.1177/036354658100900418 7258472

[3] Engel A, Feldner-Busztin H. Bilateral stress fracture of the scaphoid: a case report. Arch Orthop Trauma Surg. 1991;110:314–5. 10.1007/BF00443466 1747314

[4] Inagaki H, Inoue G. Stress fracture of the scaphoid combined with the distal radial epiphysiolysis. Br J Sports Med. 1997;31:256–7. 10.1136/bjsm.31.3.256 PMC1332537 9298566

[5] Matzkin E, Singer DI. Scaphoid stress fracture in a 13-year-old gymnast: a case report. J Hand Surg. 2000;25:710–3. 10.1053/jhsu.2000.7382 10913212

[6] Hosey RG, Hauk JM, Boland MR. Scaphoid stress fracture: an unusual cause of wrist pain in a competitive diver. Orthopedics. 2006;29:503–5. 10.3928/01477447-20060601-13 16786942

[7] Rethnam U, Yesupalan RSU, Kumar TM. Non union of scaphoid fracture in a cricketer – possibility of a stress fracture: a case report. J Med Case Rep. 2007;1:37. doi:10.1186/1752-1947-1-37. PMC1919381 17603871

[8] Yamagiwa T, Fujioka H, Okuno H, Tomatsuri M, Tsunemi K, Tanaka J, et al. Surgical treatment of stress fracture of the scaphoid of an adolescent gymnast. J Sports Sci Med. 2009;8:702–4. PMC3761527 24149614

[9] Nakamoto JC, Saito M, Medina G, Schor B. Scaphoid stress fracture in high-level gymnast: a case report. Case Rep Orthop. 2011;2011:492407. doi:10.1155/2011/492407. PMC3504270 23198214

[10] Pidemunt G, Torres-Claramunt R, Ginés A, de Zabala S, Cebamanos J. Bilateral stress fracture of the carpal scaphoid: report in a child and review of the literature. Clin J Sport Med. 2012;22:511–3. 10.1097/JSM.0b013e3182580e03 22584964

[11] Majima M, Horii E, Matsui H, Hirata H, Genda E. Load transmission through the wrist in the extended position. J Hand Surg Am. 2008;33:182–8. 10.1016/j.jhsa.2007.10.018 18294538

[12] Rettig AC. Wrist problems in the tennis players. Med Sci Sports Exerc. 1994;26:1207–12. 7799763

[13] Chow JW, Knudson DV, Tillman MD, Andrew DPS. Pre- and post-impact muscle activation in the tennis volley: effects of ball speed, ball size and side of the body. Br J Sports Med. 2007;41:754–9. 10.1136/bjsm.2007.037184 PMC2465297 17957013

